# Editorial: Capturing developmental brain dynamics

**DOI:** 10.1038/s41539-022-00126-x

**Published:** 2022-06-02

**Authors:** Milene Bonte, Nienke van Atteveldt

**Affiliations:** 1 Department of Cognitive Neuroscience, and Maastricht Brain Imaging Center, Faculty of Psychology and Neuroscience, Maastricht University, Maastricht, The Netherlands; 2 Department of Clinical Developmental Psychology & Institute Learn!, Vrije Universiteit Amsterdam, Amsterdam, The Netherlands

**Keywords:** Cognitive neuroscience, Development of the nervous system

The Collection “Capturing Developmental Brain Dynamics” results from an interdisciplinary workshop combining expertise and perspectives on changes occurring at different levels during child development.

The strongly interactive nature of the developing brain is highlighted in this Collection in the meeting report by the workshop organizers^1^ and in a methodological perspective on learning and brain development^2^. These articles explore the interactions between maturational changes in the brain (its structure, function, and connectivity) and sensory input from the environment, often called “experience”. Van Atteveldt et al.^1^ emphasize the importance of interactions between different functions (e.g., reading, math, executive functions), called interactive specialization^3^ or mutualism^4^. Van Duijvenvoorde et al.^2^ illuminate the overlap as well as differences between learning and neurobiological maturational changes. They argue that a better understanding of these processes, and how they differ, is important to inform learning interventions.

Both papers provide brief summaries of the current state of affairs, and guidance for future research to increase our understanding of how children learn and develop. Two overlapping suggested approaches are: (1) refining the use of longitudinal designs and (2) approaches to improve our understanding and analysis of interindividual variability. Regarding this first suggestion, van Duijvenvoorde et al.^2^ suggest the use of longitudinal intervention designs as a method that can potentially disentangle maturational and experience-dependent processes in the brain, if neuroimaging and behavioral data are concurrently obtained over time. The meeting report^1^ suggests that in addition to multiple timepoints and levels (such as neuroimaging and behavioral data), datasets should also include larger and more representative samples. Regarding the second suggestion, both papers point out that with the widely used analytical strategies that treat individual variability as noise, we miss meaningful types of individual variability. Several emerging approaches that enable capturing *meaningful* variability are proposed, such as complementing group-level fMRI analyses with measures of individual consistency, or clustering techniques to reveal subgroups of children that would otherwise remain hidden in the larger group. The methodological paper by Cornelizs and van Klaveren^5^ is in line with the above suggested approaches. They report a new, adaptive method of assigning individuals to intervention conditions, and thereby improve the ability to detect heterogeneous intervention effects.

Two other review papers in this Collection specifically focus on developmental brain dynamics underlying the acquisition of reading^6^ and numerical and arithmetic abilities^7^ and illustrate the importance of the methodological approaches above for research in these developmental domains. Both reading and math skills develop incrementally throughout primary school and beyond, and show large interindividual differences in learning pace and attained fluency levels. Furthermore, while most children obtain sufficient reading and math skills, 5–10% of children are faced with severe and persistent difficulties due to developmental dyslexia or dyscalculia^6,7^. As discussed in Vogel and De Smedt^7^, individual differences in math skills are formed during the interactive development of multiple brain networks subserving domain-specific (e.g., representation of numerical quantities) and domain-general functions (e.g., verbal working memory). Their review of neuroimaging studies in children and adults outlines the developmental refinement of domain-specific and domain-general brain networks for numerical representations in parietal, occipital, and temporo-parietal cortex, with an additional contribution of dorsal and ventral frontal cortex and the hippocampus for arithmetic skills. It is discussed how the impaired acquisition of numerical abilities in dyscalculia is associated with atypical development of these brain systems^7^. Crucially however, Vogel and De Smedt point out that so far, most evidence for these altered neural representations and mechanisms is based on cross-sectional studies of groups of participants in primary school age or older (e.g., adults), making it difficult to separate causes from consequences. As highlighted also in the other papers of this special issue, understanding which neurocognitive abnormalities form risk factors (causes) for a developmental learning disorder and which occur as a result of learning difficulties, requires longitudinal studies following children while they gain skill proficiency.

As reviewed by Chyl et al.^6^ a substantial number of smaller to larger scale longitudinal neuroimaging studies have been performed in the domain of reading, including studies following children with versus without family risk of dyslexia. An overview of these studies shows that typical reading development is accompanied by increased structural connectivity between visual and spoken language regions in the left hemisphere, as well as local specialization of the left ventral-occipital cortex for visual word recognition^6^. These developmental changes include non-linear, e.g., inverted-U-shaped changes, with initial increases in activation or specialization from pre-reading to early reading stages, and more focal responses with higher expertise. Longitudinal studies in children with (family risk of) dyslexia are then discussed to show both protracted development of anatomical connectivity and altered functional responses in the brain’s reading network. In studies that followed children beyond initial reading stages, these functional alterations tended to normalize again, highlighting the dynamic nature of development and the importance of longitudinal designs. Chyl et al.^6^ conclude with a relevant theme that also comes back in the papers of van Atteveldt et al.^1^ and Vogel and De Smedt^7^, that neuroimaging measures could add value to the early prediction of children’s future skills. Despite promising first results, these papers emphasize that early prediction requires more systematic longitudinal data on neurocognitive and environmental factors shaping foundational skills for reading and math prior to schooling, as well as their relation to acquired reading/math levels later on. This also brings us back to the need for improved longitudinal designs and approaches to quantify and understand interindividual variability^1,2^, with the specific addition to optimize these approaches for increased reliability and validity in this young age group.
